# NAT10 Promotes Tubular Epithelial Cell Senescence in Cisplatin-Induced Acute Kidney Injury by Regulating DDX17

**DOI:** 10.7150/ijbs.127909

**Published:** 2026-01-30

**Authors:** Yuting Zhu, Wenchao Xu, Cheng Wan, Bowen Deng, Yaru Xie, Bin Yang, Hongyang Jiang, Chun Zhang

**Affiliations:** 1 Department of Nephrology, Union Hospital, Tongji Medical College, Huazhong University of Science and Technology, Wuhan 430022, China.; 2 Department of Urology, Traditional Chinese and Western Medicine Hospital of Wuhan, Tongji Medical College, Huazhong University of Science and Technology, Wuhan 430022, China.; 3 Department of Urology, Tongji Hospital, Tongji Medical College, Huazhong University of Science and Technology, Wuhan 430030, China.

**Keywords:** NAT10, acute kidney injury, cellular senescence, DDX17, Remodelin

## Abstract

Acute kidney injury (AKI) is a severe clinical syndrome with high morbidity and mortality, yet its pathogenesis remains incompletely understood, and effective therapeutic strategies are still lacking. In this study, we observed significant upregulation of N-acetyltransferase 10 (NAT10) in the tubular epithelial cells of Cisplatin-induced AKI. Lentivirus-mediated knockdown of NAT10 or treatment with NAT10 inhibitor Remodelin, ameliorated Cisplatin-induced renal dysfunction and tubular injury. Importantly, NAT10 inhibition markedly attenuated cellular senescence in Cisplatin-induced AKI, as evidenced by reduced senescence-associated β-galactosidase (SA-β-gal) activity, downregulation of senescence markers (p53, p21 and γ-H2A.X) and decreased levels of senescence-associated secretory phenotype (SASP) factors (IL-1β, IL-6 and TNF-α). Mechanistically, co-immunoprecipitation assay suggested that NAT10 interacted with DDX17 to regulate its expression. Knockdown or inhibition of NAT10 reduced the protein expression of DDX17 in Cisplatin-injured kidneys. While silencing DDX17 could inhibit Cisplatin-induced senescence in HK-2 cells. Furthermore, we demonstrated that the effects of NAT10 on Cisplatin-induced tubular injury and senescence was dependent on DDX17. Our study revealed a novel mechanism by which NAT10 promoted Cisplatin-induced renal tubular cell senescence via DDX17 upregulation, suggesting that targeting the NAT10/DDX17 signaling axis may offer a potential therapeutic strategy for AKI.

## Introduction

Acute kidney injury (AKI) is a critical clinical syndrome mainly characterized by a rapid decline in kidney function^1^, ^2^. It represents a major global health concern associated with high morbidity, mortality and the increased risk of progression to chronic kidney disease (CKD)^1^, ^3^, ^4^. Cisplatin is a widely used antitumor chemotherapy drug, while approximate one-third of Cisplatin-treated cancer patients develop into AKI^5^, ^6^. The lack of effective therapeutic options for Cisplatin-induced AKI underscores the critical need to elucidate its underlying mechanisms for developing novel treatments.

Cellular senescence is a state of permanent cell cycle arrest that arises with age and can also be induced by stresses such as DNA damage, mitochondrial dysfunction and oncogene activation^7^, ^8^. Senescent cells remain metabolically active, secreting pro-inflammatory cytokines, chemokines, growth factors and proteases, collectively known as the senescence-associated secretory phenotype (SASP)^9^, ^10^. Compelling evidence now positions cellular senescence as a critical mechanism in the pathophysiology of Cisplatin-induced kidney injury. Studies have delineated that acute stress from Cisplatin induced premature senescence in renal tubular epithelial cells, accelerating the pathogenesis of AKI^11^, ^12^. The accumulation of senescent cells creates a pro-inflammatory and pro-fibrotic milieu via persistent SASP, which facilitates the transition from AKI to CKD^13^, ^14^. Therefore, targeting cellular senescence has emerged as a promising therapeutic strategy to mitigate AKI and prevent its detrimental long-term consequences.

N-acetyltransferase 10 (NAT10), a member of the Gcn5-related N-acetyltransferase (GNAT) family, possesses both protein and RNA acetyltransferase activities^15^, ^16^. Dysregulation of NAT10 has been implicated in the pathogenesis of diverse diseases. NAT10 overexpression stabilizes transcripts of oncogenes and proliferation-related factors via N4-acetylcytidine (ac4C) modification, driving tumorigenesis and metastasis^17^, ^18^. Genetic depletion of NAT10 or chemical inhibition via Remodelin significantly enhanced healthspan in the mouse model of human accelerated aging syndrome^19^. Furthermore, NAT10 promoted ischemia-reperfusion injury induced AKI by regulating ferroptosis and inflammation via its ac4C modification ability^20^, ^21^. On the contrary, NAT10-mediated ac4C modification played a renoprotective role in the formation of kidney stones^22^. In glomerular disease, NAT10 directly interacted with toll-like receptor 2 (TLR2) to aggravate podocyte senescence and adriamycin-induced nephropathy^23^. However, the role of NAT10 as a protein acetyltransferase in cellular senescence of renal tubular epithelial cells during AKI remained unclear. Therefore, this study aimed to elucidate the regulatory mechanism of NAT10 in Cisplatin-induced cellular senescence in AKI.

DDX17, a member of the DEAD-box RNA helicase family, is involved in various RNA metabolic processes, such as alternative splicing on pre-mRNAs, miRNA and ribosome biosynthesis, and co-regulation of transcriptional activity^24^, ^25^. Previous studies have demonstrated that DDX17 modulated multiple biological processes, including oxidative stress, autophagy, and apoptosis, and participated in immune inflammation and tumorigenesis^24^, ^26^. However, the specific role of DDX17 in kidney diseases is unknown and the association between NAT10 and DDX17 remains unclear.

In this study, we aimed to evaluate the role of NAT10 in Cisplatin-induced cellular senescence in AKI and elucidate the potential regulatory mechanisms. We found that either knockdown of NAT10 or chemical inhibition with Remodelin protected against kidney dysfunction and tubular senescence in Cisplatin-induced AKI. Mechanistically, NAT10 interacted with DDX17 to regulate its protein expression, therefore exacerbating Cisplatin-induced cellular senescence in AKI. Collectively, our work highlighted the importance of NAT10 as a potential target for alleviating cellular senescence in Cisplatin-induced AKI.

## Methods

### Animal studies

Male C57BL/6J mice aged 8 weeks and weighted 20-25 g were purchased from Charles River Co., Ltd. All animal experiments were approved by the Experimental Animal Ethics Committee of Tongji Medical College, Huazhong University of Science and Technology (IACUC Number: 4669). Mice were housed in a temperature-controlled environment under 12h/12h light-dark cycles with *ad libitum* access to food/water. For the Cisplatin-induced AKI animal model, mice were intraperitoneally injected with Cisplatin (MCE) at a dose of 25 mg/kg. The control group was intraperitoneally injected with an equivalent volume of saline. Blood and kidney tissues were collected at 1, 2 and 3 days post-injection, respectively. For the AKI model with intrarenal lentivirus delivery, NAT10 knockdown lentivirus (shNAT10) or negative control lentivirus (shNC) was administered via renal cortex injection. Briefly, each mouse was injected with 1 × 10^6^ TU/ml lentivirus (shNAT10 or shNC) at 5 different sites (10 ul/site) 7 days prior to Cisplatin injection. For the AKI model treated with Remodelin (MCE), Remodelin (20 mg/kg) was injected intraperitoneally on the second day after injection of Cisplatin. Remodelin was dissolved in a solution containing 5% DMSO, 40% PEG300, 5% Tween-80 and 50% ddH_2_O to prepare a working solution with a concentration of 2.5 mg/mL. After three consecutive days of Remodelin administration, blood and kidney tissues were collected from the mice. For the AKI model with preventive treatment of Remodelin, Remodelin was injected intraperitoneally for three consecutive days before Cisplatin administration. The second day post-Cisplatin injection, Remodelin was administered for another three consecutive days. Subsequently, blood and kidney tissues were collected from the mice. For the safety evaluation experiment of Remodelin, mice were intraperitoneally injected with 20 mg/kg/d Remodelin for 30 consecutive days, while the other two groups were injected with an equal amount of saline or vehicle respectively.

### Serum biochemistry tests

Renal function parameters including serum creatinine and blood urea nitrogen were measured with a Creatinine Assay Kit (DICT-500, Bioassay Systems). And serum aspartate aminotransferase (AST) and alanine aminotransferase (ALT) were measured using an automatic biochemical analyzer.

### Cell culture and treatment

Human proximal tubule epithelial (HK-2) line was purchased from American Type Culture Collection. HK-2 cells were cultured in DMEM/F12 medium (Gibco) supplemented with 10% fetal bovine serum (VivaCell) at 37 °C under 5% CO₂. In the concentration gradient experiment, cells were exposed to 0, 5, 10 and 20 μM Cisplatin for 24 hours. In the time gradient experiment, cells were exposed to 20 μM Cisplatin for 0, 6, 12 and 24 hours. Cisplatin stimulation for 24 hours at 20 μM concentration was selected for subsequent experiments. For lentivirus-mediated gene knockdown experiments, HK-2 cells were seeded in a 6-well plate at 20-30% confluence and transfected with shNC or shNAT10 lentivirus at an MOI of 10. After 12 hours, the medium was refreshed. GFP expression was examined at 72 hours post-transfection, followed by selection with 2 μg/mL puromycin (Beyotime) to remove untransfected cells. HK-2 cells were treated with 5 nM Remodelin concurrently with Cisplatin for 24 hours before sample collection. For siRNA experiments, HK-2 cells were transfected with negative control siRNA (NC-si) or DDX17 siRNA (DDX17-si) using Lipofectamine 2000 (Thermo Fisher Scientific), followed by Cisplatin exposure for 24 hours. For plasmid experiments, HK-2 cells were transfected with NAT10 overexpression plasmids using Lipofectamine 2000, followed by Cisplatin and 1 μM Ruxolitinib (MCE) treatment for 24 hours. To evaluate protein stability, HK-2 cells were treated with cycloheximide (MCE) for 0, 4 and 8 hours after 24-hour Cisplatin exposure, followed by sample collection for degradation analysis.

### Western blotting

Kidney tissues and cultured cells were lysed in RIPA lysis buffer containing PMSF, phosphatase inhibitors and protease inhibitors. The protein concentration was determined using the BCA assay kit. Proteins were then separated by SDS-PAGE and electrophoretically transferred to PVDF membranes (Millipore). Membranes were blocked with 5% non-fat milk for 1 hour at room temperature, followed by overnight incubation with primary antibodies at 4°C. The primary antibodies were listed below: NAT10 (1:2000, 13365-1-AP, Proteintech), NGAL (1:1000, ab63929, Abcam), β-actin (1:5000, 20536-1-AP, Proteintech), KIM-1 (1:1000, 14971, Cell Signaling Technology), GFP (1:200, sc-9996, Santa Cruz Biotechnology), p53 (1:1000, 10442-1-AP, Proteintech), p16 (1:1000, 10883-1-AP, Proteintech), γ-H2A.X (1:1000, 9718S, Cell Signaling Technology), p21 (1:200, sc-6246, Santa Cruz Biotechnology), DDX17 (1:1000, 19910-1-AP, Proteintech) and TLR2 (1:1000, 66645-1-Ig, Proteintech). Subsequently, membranes were incubated with horseradish peroxidase conjugated secondary antibodies for 1 hour at room temperature. Protein bands were visualized using ECL Western Blotting Substrate (Abbkine) and imaged with a BioSpectrum Imaging System (UVP). Quantification analysis was carried out using ImageJ software (NIH).

### Real-time quantitative PCR analysis

RNA extraction, cDNA synthesis, and real-time quantitative PCR were performed according to previous protocols^27^. The primer sequences were listed in Table S1.

### Renal histology and immunohistochemistry

Kidney samples fixed in 4% paraformaldehyde were processed into 3 μm paraffin sections and subjected to hematoxylin and eosin (H&E) staining for histological evaluation. Tubular injury was evaluated semiquantitatively (scale 0-4) based on the presence of tubular dilation, cast deposition, brush border loss and epithelial cell detachment. The scoring criteria were described below: 0 (normal), 1 (<25%), 2 (25-50%), 3 (51-75%) and 4 (>75%)^11^, ^28^.

The immunohistochemistry was performed according to previous protocols^27^, ^29^. The primary antibodies for NAT10 (1:200, Proteintech), NGAL (1:200, Abcam), γ-H2A.X (1:200, Cell Signaling Technology) and IL-1β (1:200, 12242, Cell Signaling Technology) were used.

### Immunofluorescent staining

The immunofluorescent staining was performed according to previous protocols^27^, ^30^. The primary antibodies for NAT10 (1:200, Proteintech), F4/80 (1:200, A23788, ABclonal) and γ-H2A.X (1:200, Cell Signaling Technology) were used. To better define NAT10 expression in the proximal tubule, NAT10 was co-stained with proximal tubule specific marker lotus tetragonolobus lectin (LTL) (1:50, Thermo Fisher Scientific).

### RNA-sequencing analysis

Total RNAs were extracted from Cisplatin-induced HK-2 cells transfected with either negative control siRNA or NAT10 siRNA using RNAiso Plus reagent (Takara). RNA-seq experiment and data analysis were performed by Seqhealth Technology Co., LTD (Wuhan, China). Stranded RNA-seq libraries were constructed from 2 μg total RNA with the KC-Digital™ Stranded mRNA Library Prep Kit (Seqhealth), which incorporates UMIs (8 random bases) during first-strand cDNA synthesis to correct for PCR and sequencing duplicates. The library products (200-500 bps) were enriched, quantified and finally sequenced on DNBSEQ-T7 sequencer (MGI Tech Co., Ltd. China) with PE150 model. Reads were aligned to the reference genome and gene-level counts were obtained using featureCounts (Subread-1.5.1, Bioconductor). Gene expression was normalized as RPKM. Differential expression analysis was conducted with edgeR (v3.12.1), and functional enrichment analysis of differentially expressed genes was performed using KOBAS (v2.1.1). The data of RNA-seq analysis was provided in Table S2.

### SA-β-gal staining

The SA-β-gal activity was assessed according to manufacturer's protocol (Solarbio) for both 5-μm-thick frozen kidney sections and cells grown on coverslips. Subsequently, images were acquired, and the percentage of SA-β-gal positive cells or area was quantified using ImageJ software.

### Reactive oxygen species detection

The levels of reactive oxygen species in kidney sections were measured using the fluorescent probe Dihydroethidium (DHE, Beyotime). Kidney sections were incubated with DHE probe for 60 minutes at 37 °C, followed by DAPI counterstaining to visualize nuclei. Fluorescence images were acquired using a microscope for subsequent analysis.

### LC-MS/MS analysis

Beads samples from kidney cortex were incubated, digested and centrifugated to obtain peptide samples. All samples were processed by an UltiMate 3000 RSLCnano system coupled online to a Q Exactive HF mass spectrometer equipped with a Nanospray Flex ion source (Thermo). Mass spectrometry (MS) was performed in data-dependent acquisition mode, selecting the top 20 precursors for fragmentation from a full-scan range of 350-1500 m/z. Full MS scans were acquired at a resolution of 60,000 (at m/z 200) with an AGC target of 3×10^6^ and a maximum injection time of 30 ms. Isolation windows of 1.4 m/z were used, and fragmentation was performed via higher-energy collisional dissociation at 28% normalized collision energy. MS/MS scans were recorded at a resolution of 15,000, with an AGC target of 1×10^5^ and a maximum fill time of 50 ms. Dynamic exclusion was set to 30 seconds. Raw MS data were processed using MaxQuant with the Andromeda search engine^31^, ^32^. Spectra were queried against the Mouse protein database (2023-01-03) with the following parameters: tryptic digestion allowing up to two missed cleavages; fixed modification of carbamidomethylation (C); variable modifications of oxidation (M), N-terminal acetylation, and deamidation (N/Q); MS1 mass tolerances of 20 ppm (first search) and 4.5 ppm (main search); and MS2 mass tolerance of 20 ppm. Label-free quantification was enabled. Identifications were filtered to achieve a false discovery rate (FDR) of 1% at both the protein and peptide levels. Proteins categorized as decoy hits, contaminants, or identified only by removed sites were excluded from downstream quantitative analysis.

### Co-immunoprecipitation (Co-IP)

HK-2 cells were lysed in IP lysis buffer (Beyotime) supplemented with 1× protease inhibitor and PMSF for 30 minutes and then centrifuged to obtain the supernatants. 20 μL aliquot of the supernatant was reserved as input. The remaining supernatant was incubated with Protein A/G Magnetic Beads (MCE) and 2.5 μg of either specific primary antibody or rabbit IgG at 4 °C overnight with rotation. After washing with ice-cold PBS, the immunoprecipitated proteins were eluted and denatured in loading buffer at 100 °C for subsequent immunoblotting analysis.

### Statistical analysis

Data were presented as mean ± SEM. Group comparisons were performed using two-tailed unpaired Student's t-test for two groups or one-way ANOVA with Tukey's post hoc test for multiple groups. All tests were two-sided, with *p* < 0.05 considered statistically significant. All analyses were conducted and visualized using GraphPad Prism 9.0.

## Results

### NAT10 is upregulated in mice and HK-2 cells with Cisplatin-induced AKI

**Figure 1:**
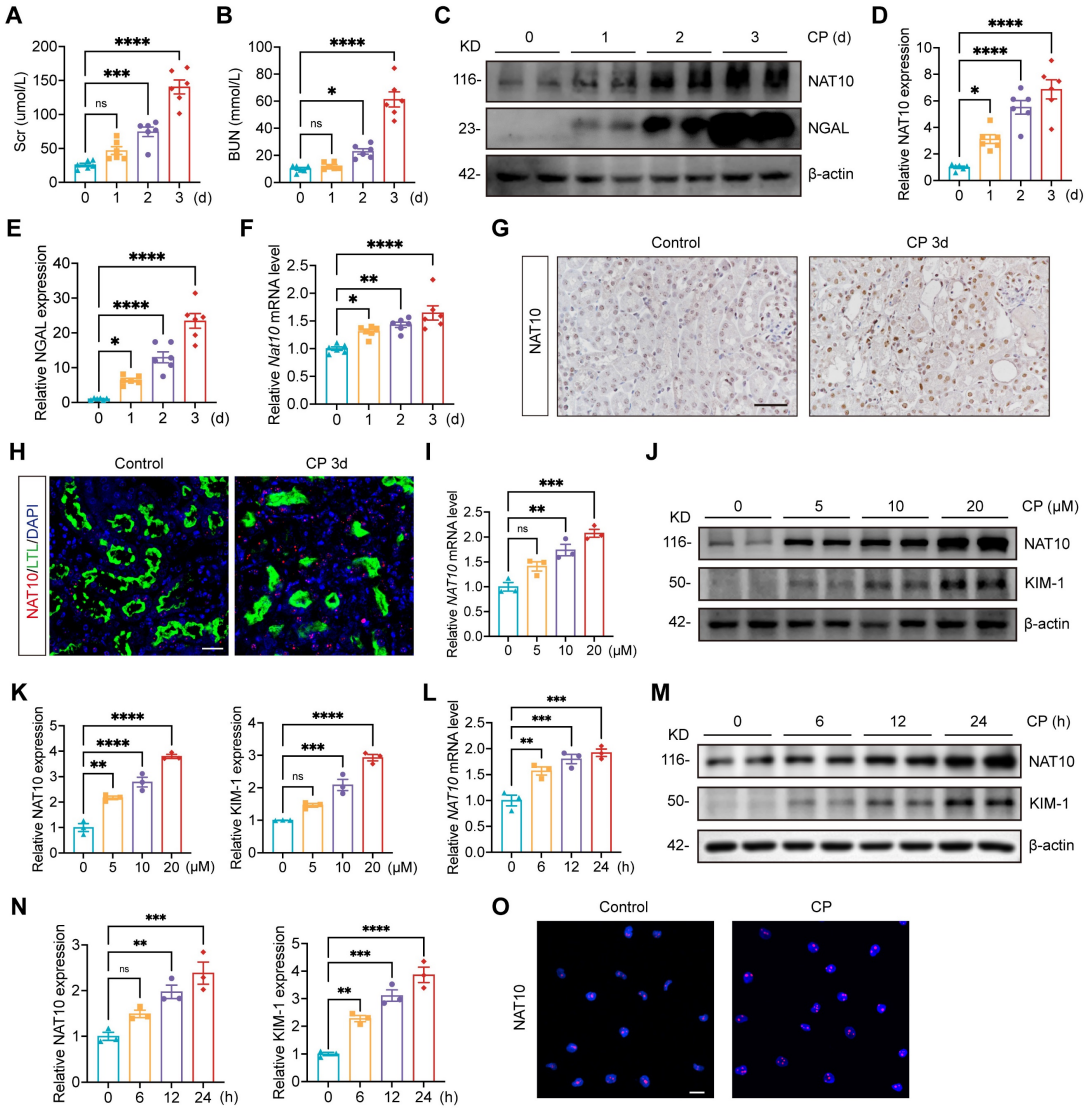
** NAT10 expression was increased in Cisplatin-induced AKI. (**A and B) Serum creatinine (Scr) (A) and blood urea nitrogen (BUN) (B) in mice treated with Cisplatin for 0, 1, 2 and 3 days (n=6). (C-E) Representative western blot images and quantification of NAT10 and NGAL in the kidney cortex (n=6). (F) qPCR analysis of *Nat10* in the kidney cortex (n=6). (G) Representative immunohistochemical staining images of NAT10 in the kidney of control and Cisplatin-treated mice. Scale bar: 50 µm. (H) Representative immunofluorescence (IF) co-staining images of NAT10 and lotus tetragonolobus lectin (LTL) in the kidney. Scale bar: 25 µm. (I) qPCR analysis of *NAT10* in HK-2 cells treated with 0, 5, 10 and 20 μM Cisplatin (n=3). (J and K) Representative western blot images and quantification of NAT10 and KIM-1 in HK-2 cells (n=3). (L) qPCR analysis of *NAT10* in HK-2 cells exposed to 20 μM Cisplatin for 0, 6, 12 and 24 hours (n=3). (M and N) Representative western blot images and quantification of NAT10 and KIM-1 in HK-2 cells (n=3). (O) Representative IF staining images of NAT10 in control and Cisplatin-treated HK-2 cells. Scale bar: 20 µm. Data were mean ± SEM. *P < 0.05, **P < 0.01, ***P < 0.001 and ****P < 0.0001. ns, not significant. One-way ANOVA followed by Tukey's post-test (A, B, D-F, I, K, L and N).

To investigate the expression pattern of NAT10 in Cisplatin-induced AKI, we utilized Cisplatin-induced mouse and tubular epithelial cell models. Serum creatinine (Scr) and blood urea nitrogen (BUN) levels were significantly elevated on day 2 post-Cisplatin administration and peaked on day 3 (Fig. 1A and 1B). Elevated protein expression of NGAL confirmed notable renal damage following Cisplatin treatment (Fig. 1C and 1E). Both mRNA and protein expression levels of NAT10 were increased in the renal cortex as early as day 1 after Cisplatin exposure and reached their maximum on day 3 (Fig. 1C, 1D and 1F). Immunohistochemical (IHC) staining further supported these findings and revealed that NAT10 was localized in the nucleus (Fig. 1G). Immunofluorescence (IF) co-staining of NAT10 and lotus tetragonolobus lectin (LTL) demonstrated upregulated NAT10 expression in proximal tubular epithelial cells after Cisplatin treatment (Fig. 1H).

To validate the *in vivo* results, HK-2 cells were treated with 0, 5, 10 and 20 μM Cisplatin. The mRNA and protein levels of NAT10 were upregulated and peaked at 20 μM Cisplatin stimulation (Fig. 1I-K). And HK-2 cells exposed to Cisplatin for varying durations also resulted in an upregulation of NAT10 expression, which peaked at 24 hours (Fig. 1L-N). IF staining confirmed the predominant nuclear expression of NAT10 (Fig. 1O).

### Lentivirus-mediated NAT10 knockdown attenuated Cisplatin-induced AKI

**Figure 2:**
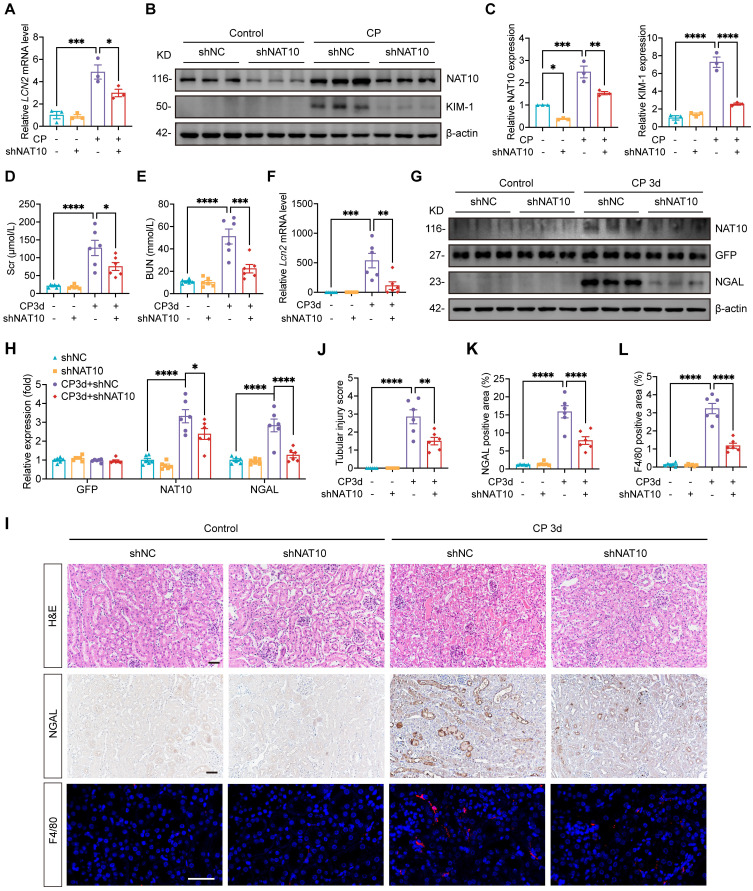
** Lentivirus mediated NAT10 knockdown attenuated Cisplatin-induced AKI. (**A) qPCR analysis of *LCN2* in Cisplatin treated HK-2 cells transfected with negative control lentivirus (shNC) or NAT10 knockdown lentivirus (shNAT10) (n=3). (B and C) Representative western blot images and quantification of NAT10 and KIM-1 in HK-2 cells (n=3). (D and E) Serum creatinine (Scr) (D) and blood urea nitrogen (BUN) (E) in Cisplatin-induced mice with kidney cortex injection of shNC or shNAT10 lentivirus (n=6). (F) qPCR analysis of* Lcn2* in the kidney cortex (n=6). (G and H) Representative western blot images and quantification of NAT10, GFP and NGAL in the kidney cortex (n=6). (I) Representative hematoxylin and eosin (H&E) staining images (upper panel), immunohistochemical staining images of NGAL (middle panel) and immunofluorescence staining images of F4/80 (lower panel) in the kidney. Scale bar: 50 µm. (J) Tubular injury scores were calculated according to H&E staining images (n=6). (K) The percentages of NGAL positive area were calculated (n=6). (L) The percentages of F4/80 positive area were calculated (n=6). Data were mean ± SEM. *P < 0.05, **P < 0.01, ***P < 0.001 and ****P < 0.0001. One-way ANOVA followed by Tukey's post-test (A, C-F, H and J-L).

To further investigate the role of NAT10 in AKI, HK-2 cells were transfected with NAT10 knockdown lentivirus prior to Cisplatin exposure. Knockdown of NAT10 effectively attenuated Cisplatin-induced damage in HK-2 cells as revealed by the levels of *LCN2* and KIM-1 (Fig. 2A-C). These findings indicated that knockdown of NAT10 exhibited protection against Cisplatin-induced injury in HK-2 cells.

To validate these findings *in vivo*, lentivirus-mediated knockdown of NAT10 was delivered via injection in kidney cortex. Scr and BUN levels indicated that NAT10 knockdown improved kidney function in Cisplatin-treated mice (Fig. 2D and 2E). Western blot analysis of NAT10 and GFP confirmed efficient knockdown of NAT10 in the kidney cortex (Fig. 2G and 2H). Additionally, mRNA and protein levels of NGAL revealed a significant reduction in the injury of renal tubular epithelial cells (Fig. 2F-I and 2K). Hematoxylin and eosin (H&E) staining illustrated that NAT10 knockdown ameliorated Cisplatin-induced pathological changes in the kidney (Fig. 2I and 2J). Moreover, IF staining of F4/80 showed that NAT10 knockdown reduced the infiltration of macrophages in the kidney (Fig. 2I and 2L). Collectively, these results demonstrated that knockdown of NAT10 alleviated Cisplatin-induced acute renal epithelial cell damage.

### Treatment with NAT10 inhibitor Remodelin alleviated Cisplatin-induced AKI

**Figure 3:**
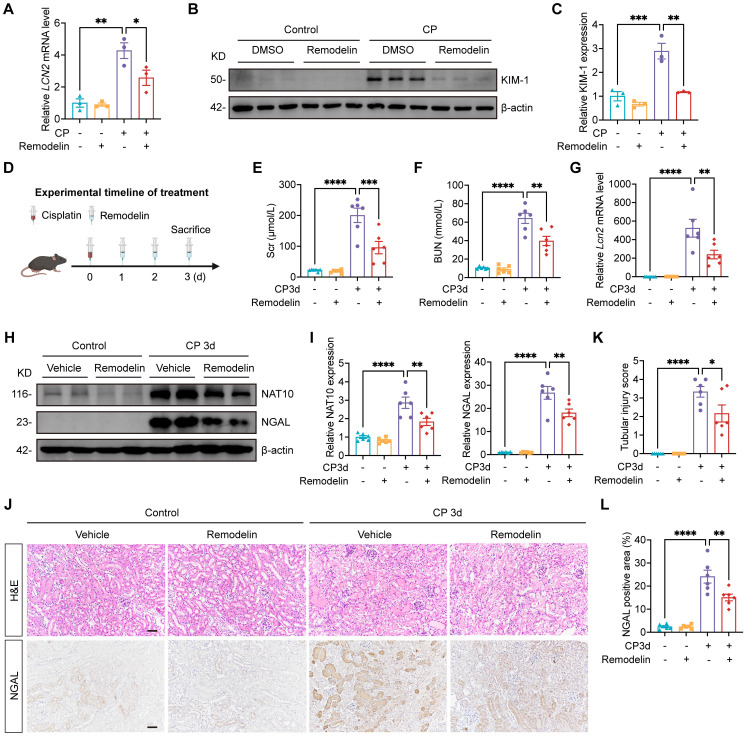
** Treatment with NAT10 inhibitor Remodelin alleviated Cisplatin-induced AKI.** (A) qPCR analysis of *LCN2* in Cisplatin-induced HK-2 cells treated with DMSO or Remodelin (n=3). (B and C) Representative western blot images and quantification of KIM-1 in HK-2 cells (n=3). (D) Schematic diagram of Remodelin treatment in Cisplatin-induced AKI. (E and F) Serum creatinine (Scr) (E) and blood urea nitrogen (BUN) (F) in mice (n=6). (G) qPCR analysis of* Lcn2* in the kidney cortex (n=6). (H and I) Representative western blot images and quantification of NAT10 and NGAL in the kidney cortex (n=6). (J) Representative hematoxylin and eosin (H&E) staining images (upper panel) and immunohistochemical staining images of NGAL (lower panel) in the kidney. Scale bar: 50 µm. (K) Tubular injury scores were calculated according to H&E staining images (n=6). (L) The percentages of NGAL positive area were calculated (n=6). Data were mean ± SEM. *P < 0.05, **P < 0.01, ***P < 0.001 and ****P < 0.0001. One-way ANOVA followed by Tukey's post-test (A, C, E-G, I, K and L).

Having established that renal NAT10 knockdown alleviated Cisplatin-induced AKI, we next evaluated the therapeutic potential of NAT10 inhibitor Remodelin. In Cisplatin-induced HK-2 cells, Remodelin downregulated the levels of *LCN2* and KIM-1 (Fig. 3A-C). Then the model for Remodelin treatment in Cisplatin-induced AKI was constructed in mice (Fig. 3D). Assessment of kidney function revealed that Remodelin treatment significantly attenuated Cisplatin-induced elevation in Scr and BUN (Fig. 3E and 3F). Furthermore, both qPCR and Western blot analyses demonstrated that Remodelin effectively reduced the expression of NGAL, a biomarker of tubular injury. IHC analysis further confirmed the reduction in renal NGAL expression following Remodelin treatment (Fig. 3G-J and 3L). H&E staining and tubular injury scores indicated that Remodelin ameliorated Cisplatin-induced tubular necrosis, dilation, and cast formation (Fig. 3J and 3K). Together, these results demonstrated that *in vivo* inhibition of NAT10 with Remodelin improved kidney function and mitigated kidney injury in Cisplatin-treated mice.

To assess the safety of Remodelin, mice were administered Remodelin continuously for one month (Fig. S1A). Scr, BUN, AST and ALT showed no significant differences among the saline, vehicle, and Remodelin treatment groups (Fig. S1B and S1C). Moreover, gross morphological examination of the liver, spleen, heart, and kidneys revealed no apparent abnormalities across the three groups (Fig. S1D). Additionally, the organ-to-body weight ratios of the liver and spleen did not differ significantly among the groups (Fig. S1E). Collectively, these results demonstrated that Remodelin administration was well-tolerated and exhibited no detectable toxicity in mice.

### NAT10 accelerated Cisplatin-induced cellular senescence in HK-2 cells

**Figure 4:**
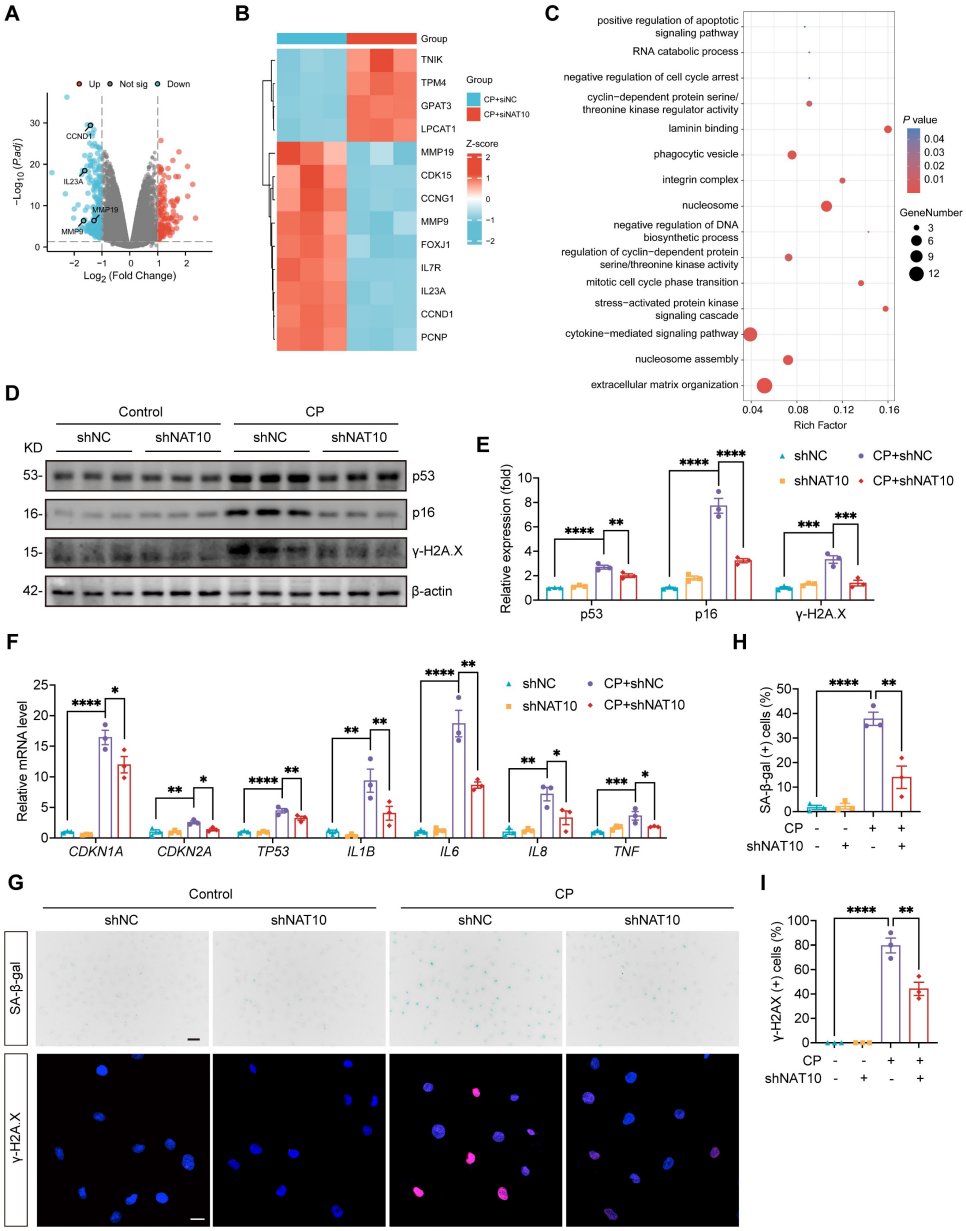
** NAT10 accelerated Cisplatin-induced cellular senescence in HK-2 cells.** (A) A volcano plot of differentially expressed genes (DEGs) in Cisplatin-induced HK-2 cells transfected with negative control siRNA or NAT10 siRNA. (B) Heatmap of DEGs associated with senescence. (C) GO enrichment analysis of DEGs. (D and E) Representative western blot images and quantification of p53, p16 and γ-H2A.X in Cisplatin treated HK-2 cells transfected with negative control lentivirus (shNC) or NAT10 knockdown lentivirus (shNAT10) (n=3). (F) qPCR analysis of *CDKN1A*, *CDKN2A*, *TP53*, *IL1B*, *IL6*, *IL8* and *TNF* in HK-2 cells (n=3). (G) Representative SA-β-gal staining images (upper panel) and immunofluorescence staining images of γ-H2A.X (lower panel) in HK-2 cells. Scale bar: 50 µm (upper panel) and 20 µm (lower panel). (H) The percentages of SA-β-gal positive cells were calculated (n=3). (I) The percentages of γ-H2A.X positive cells were calculated (n=3). Data were mean ± SEM. *P < 0.05, **P < 0.01, ***P < 0.001 and ****P < 0.0001. One-way ANOVA followed by Tukey's post-test (E, F, H and I).

To elucidate the specific regulatory mechanisms of NAT10 in Cisplatin-induced tubular epithelial cell injury, we transfected Cisplatin-induced HK-2 cells with either negative control siRNA or NAT10 siRNA and performed RNA sequencing. Volcano plot and heat map revealed numerous differentially expressed genes (DEGs) between the two groups. Notably, senescence-associated genes were markedly downregulated upon NAT10 silencing (Fig. 4A and 4B). GO enrichment analysis of DEGs highlighted significant enrichment in terms including “negative regulation of cell cycle arrest” and “cytokine-mediated signaling pathway” (Fig. 4C). Given that senescent cells typically exhibit cell cycle arrest and abundant cytokine secretion, these findings suggested that NAT10 silencing may suppress Cisplatin-induced senescence in HK-2 cells. Cisplatin stimulation significantly increased the expression of senescence markers p53, p21 and p16 in HK-2 cells; however, these changes were attenuated by NAT10 knockdown (Fig. 4D-F). Similarly, the mRNA levels of SASP factors, including *IL1B*, *IL6*, *IL8* and *TNF*, were markedly elevated following Cisplatin exposure, and these increases were significantly reduced upon NAT10 knockdown (Fig. 4F). In addition, Cisplatin induced a pronounced elevation in the DNA damage marker phosphorylated histone H2A.X (γ-H2A.X), which was effectively suppressed by NAT10 knockdown (Fig. 4D and 4E). This reduction in γ-H2A.X was further confirmed by IF staining (Fig. 4G and 4I). Furthermore, Cisplatin induction resulted in a substantial increase in SA-β-gal positive senescent cells, and NAT10 knockdown significantly decreased the proportion of these senescent cells (Fig. 4G and 4H).

### NAT10 knockdown improved cellular senescence in the kidney of Cisplatin-induced AKI

**Figure 5:**
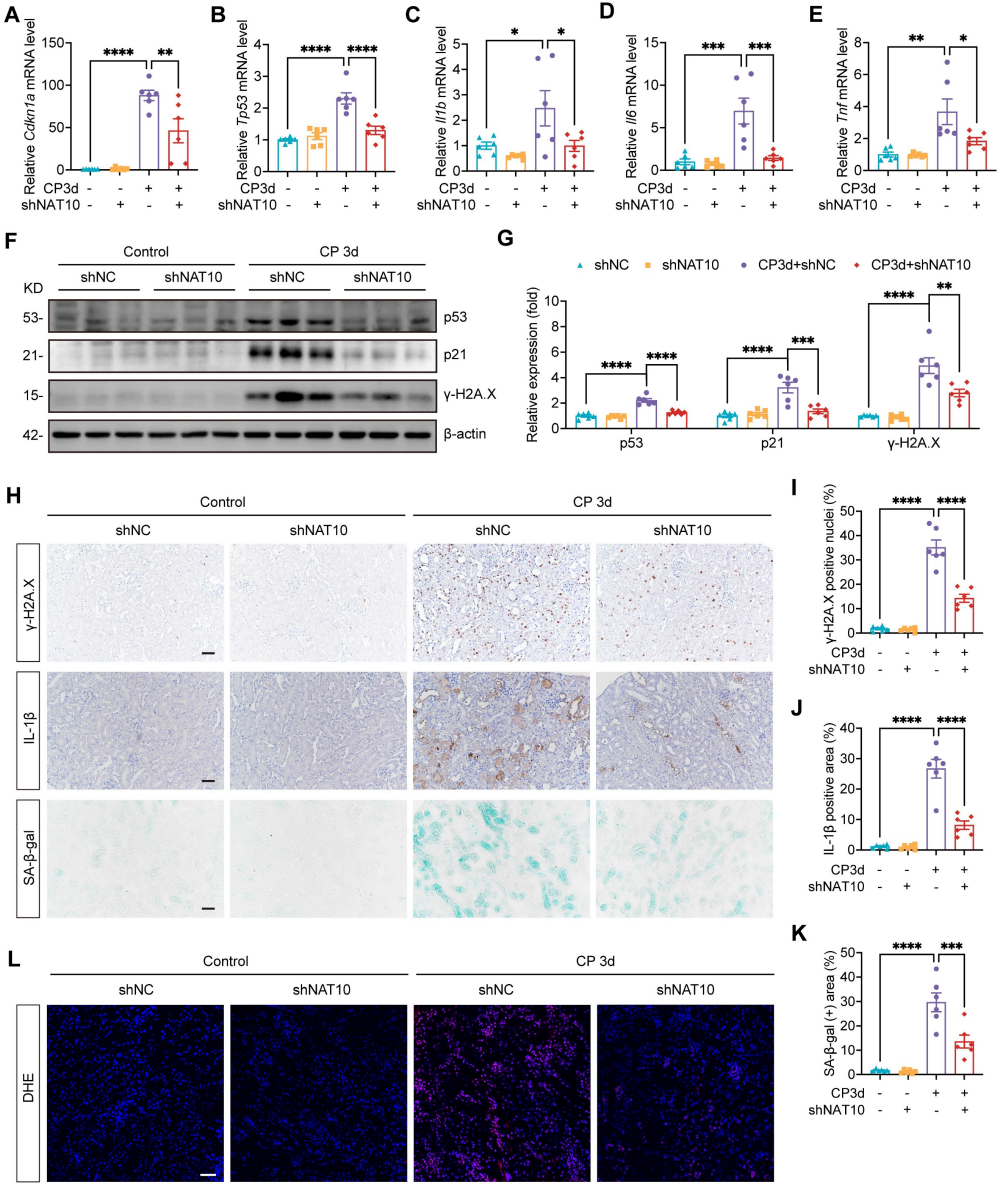
** NAT10 knockdown improved cellular senescence in the kidney of Cisplatin-induced AKI.** (A-E) qPCR analysis of *Cdkn1a* (A), *Tp53* (B), *Il1b* (C), *Il6* (D) and *Tnf* (E) in the kidney cortex of Cisplatin-induced mice with kidney cortex injection of negative control lentivirus (shNC) or NAT10 knockdown lentivirus (shNAT10) (n=6). (F and G) Representative western blot images and quantification of p53, p21 and γ-H2A.X in the kidney cortex (n=6). (H) Representative immunohistochemical staining images of γ-H2A.X (upper panel) and IL-1β (middle panel), and SA-β-gal staining images (lower panel) in the kidney. Scale bar: 50 µm. (I) The percentages of γ-H2A.X positive nuclei were calculated (n=6). (J) The percentages of IL-1β positive area were calculated (n=6). (K) The percentages of SA-β-gal positive area were calculated (n=6). (L) Representative DHE staining images of the kidney. Data were mean ± SEM. *P < 0.05, **P < 0.01, ***P < 0.001 and ****P < 0.0001. One-way ANOVA followed by Tukey's post-test (A-E, G and I-K).

To validate the role of NAT10 in Cisplatin-induced cellular senescence *in vivo*, we evaluated cellular senescence in the kidney of AKI model with NAT10 knockdown. Consistent with previous studies, Cisplatin stimulation elevated the expression of senescence markers p53 and p21 in the kidney, which was attenuated by NAT10 knockdown (Fig. 5A, 5B, 5F and 5G). The mRNA levels of SASP factors were significant increase in the kidney of AKI mice, with IHC staining further confirming the elevated protein level of IL-1β. However, NAT10 knockdown reduced the level of SASP in the kidney of AKI mice (Fig. 5C-E, 5H and 5J). Additionally, NAT10-knockdown AKI mice showed a decreased level of γ-H2A.X and a reduced proportion of γ-H2A.X positive nuclei in the kidney (Fig. 5F-I). Cisplatin also resulted in elevated expression of SA-β-gal in the kidney, which was significantly mitigated by NAT10 knockdown (Fig. 5H and 5K). Given the pivotal role of oxidative stress in cellular senescence, we further assessed the effect of NAT10 on oxidative stress. Dihydroethidium (DHE) staining demonstrated that NAT10 knockdown alleviated Cisplatin-induced oxidative stress in the kidney (Fig. 5L), suggesting that the regulation of cellular senescence by NAT10 may be closely related to its impact on oxidative stress.

Both *in vivo* and *in vitro* experiments have confirmed that NAT10 knockdown alleviated cellular senescence in Cisplatin-induced AKI. However, the specific role of NAT10-mediated senescence in AKI pathogenesis remained unclear. To address this, we overexpressed NAT10 in Cisplatin-treated HK-2 cells via plasmids transfection and intervened with the senomorphic drug Ruxolitinib^7^, ^33^, ^34^. The results demonstrated that NAT10 overexpression exacerbated Cisplatin-induced cellular injury and senescence in HK-2 cells, while Ruxolitinib treatment ameliorated the damage and senescence caused by NAT10 overexpression (Fig. S2). Therefore, NAT10 promoted AKI, at least in part, by driving tubular epithelial cell senescence.

### Treatment with NAT10 inhibitor Remodelin alleviated cellular senescence in HK-2 cells and the kidney of Cisplatin-induced AKI

**Figure 6:**
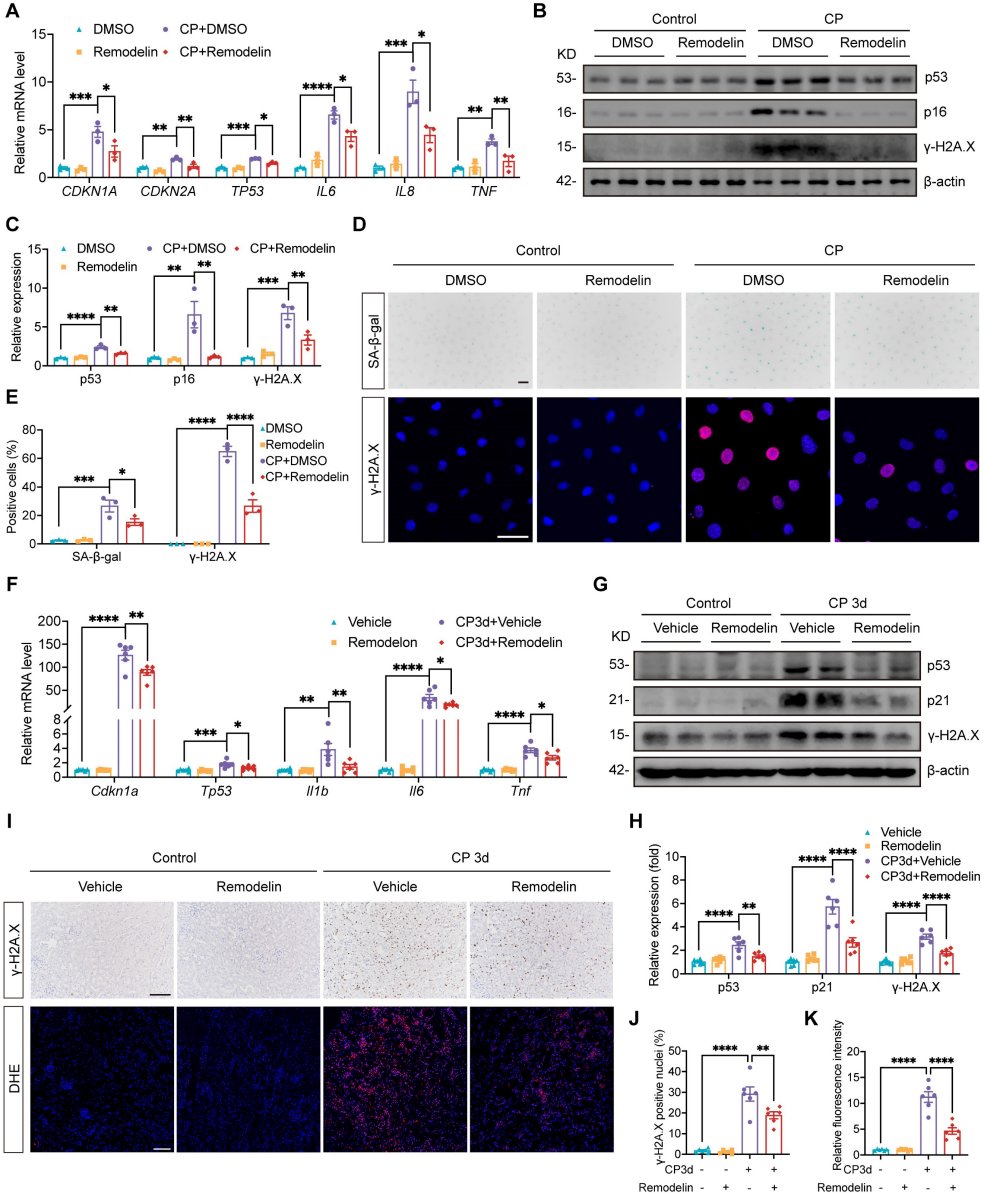
** Treatment with NAT10 inhibitor Remodelin attenuated cellular senescence in HK-2 cells and the kidney of Cisplatin-induced AKI.** (A) qPCR analysis of *CDKN1A*, *CDKN2A*, *TP53*, *IL6*, *IL8* and *TNF* in Cisplatin-induced HK-2 cells treated with DMSO or Remodelin (n=3). (B and C) Representative western blot images and quantification of p53, p16 and γ-H2A.X in HK-2 cells (n=3). (D) Representative SA-β-gal staining images (upper panel) and immunofluorescence staining images of γ-H2A.X (lower panel) in HK-2 cells. Scale bar: 50 µm. (E) The percentages of SA-β-gal positive cells and γ-H2A.X positive cells were calculated (n=3). (F) qPCR analysis of* Cdkn1a*, *Tp53*, *Il1b*, *Il6* and *Tnf* in the kidney cortex of Cisplatin-induced mice treated with vehicle or Remodelin (n=6). (G and H) Representative western blot images and quantification of p53, p21 and γ-H2A.X in the kidney cortex (n=6). (I) Representative immunohistochemical staining images of γ-H2A.X (upper panel) and DHE staining images (lower panel) in the kidney. Scale bar: 100 µm. (J) The percentages of γ-H2A.X positive nuclei were calculated (n=6). (K) Relative fluorescence intensity of DHE was calculated (n=6). Data were mean ± SEM. *P < 0.05, **P < 0.01, ***P < 0.001 and ****P < 0.0001. One-way ANOVA followed by Tukey's post-test (A, C, E, F, H, J and K).

Considering the protective effects of Remodelin on Cisplatin-induced AKI, we further investigated its potential therapeutic effects on Cisplatin-induced cellular senescence. In HK-2 cells exposed to Cisplatin, Remodelin produced therapeutic effects similar to those observed with NAT10 knockdown. Specifically, Remodelin attenuated Cisplatin-induced upregulation of senescence markers p53, p21 and p16 and suppressed the expression of SASP factors *IL6*, *IL8* and *TNF* (Fig. 6A-C). Moreover, Remodelin reduced the level of γ-H2A.X and the proportion of SA-β-gal positive HK-2 cells (Fig. 6B-E). *In vivo*, Remodelin treatment significantly reduced the mRNA levels of SASP factors (Fig. 6F). In addition, the expression of senescence markers p53 and p21 was notably downregulated following Remodelin administration (Fig. 6F-H). Western blot and IHC analyses revealed a marked reduction in renal DNA damage, as indicated by decreased γ-H2A.X level in the kidney of Remodelin-treated AKI mice (Fig. 6G-J). Furthermore, DHE staining showed that Remodelin alleviated Cisplatin-induced renal oxidative stress (Fig. 6I and 6K). In summary, these results indicated that Remodelin treatment attenuated cellular senescence in HK-2 cells and the kidney of Cisplatin-induced AKI.

### Prevention treatment with NAT10 inhibitor Remodelin attenuated Cisplatin-induced AKI and cellular senescence

**Figure 7:**
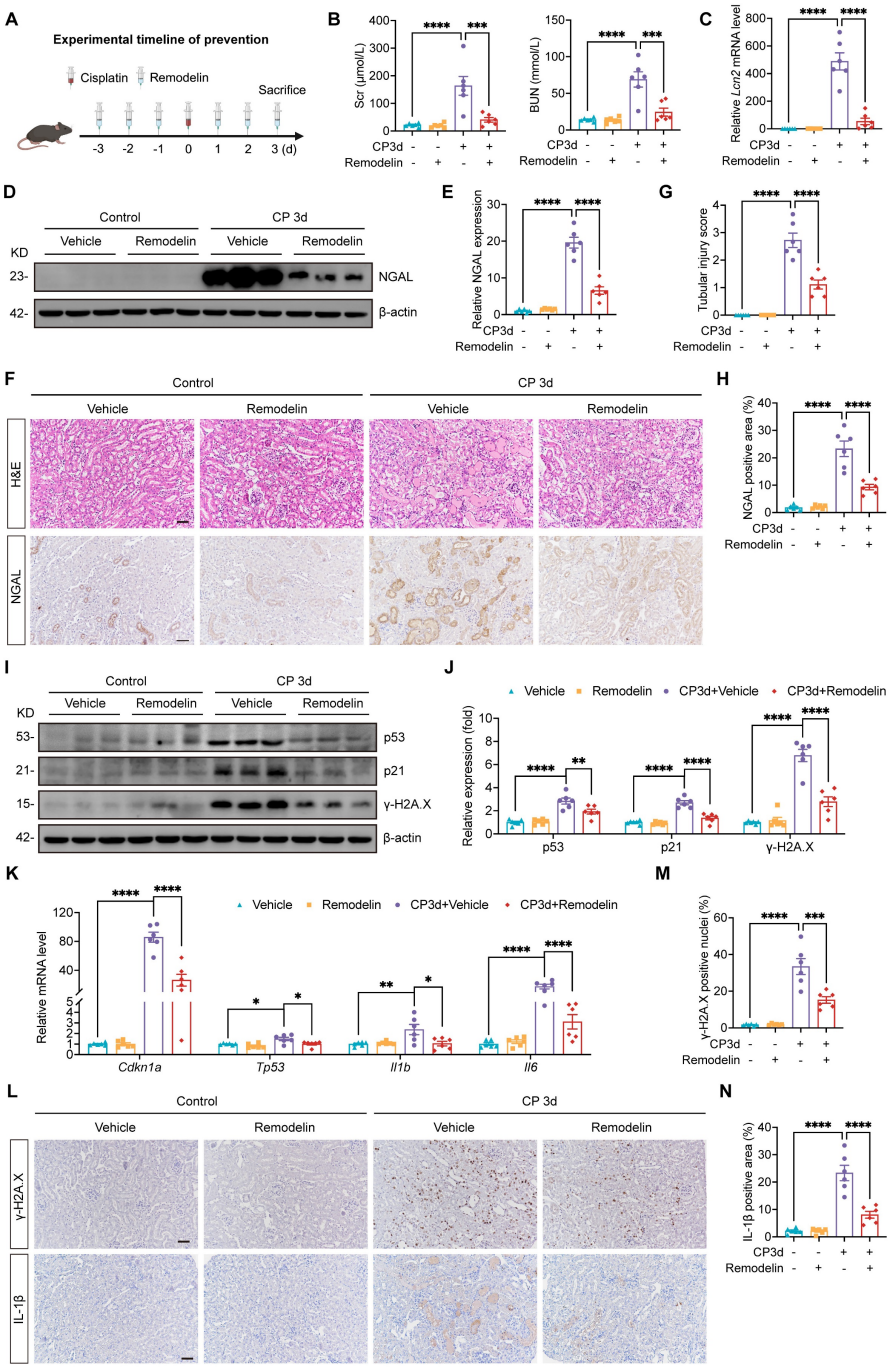
** Prevention treatment with NAT10 inhibitor Remodelin attenuated Cisplatin-induced AKI and cellular senescence.** (A) Schematic diagram of prevention treatment of Remodelin in Cisplatin-induced AKI. (B) Serum creatinine (Scr) and blood urea nitrogen (BUN) in mice (n=6). (C) qPCR analysis of* Lcn2* in the kidney cortex (n=6). (D and E) Representative western blot images and quantification of NGAL in the kidney cortex (n=6). (F) Representative hematoxylin and eosin (H&E) staining images (upper panel) and immunohistochemical (IHC) staining images of NGAL (lower panel) in the kidney. Scale bar: 50 µm. (G) Tubular injury scores were calculated according to H&E staining images (n=6). (H) The percentages of NGAL positive area were calculated (n=6). (I and J) Representative western blot images and quantification of p53, p21 and γ-H2A.X in the kidney cortex (n=6). (K) qPCR analysis of* Cdkn1a*, *Tp53*, *Il1b* and *Il6* in the kidney cortex (n=6). (L) Representative IHC staining images of γ-H2A.X (upper panel) and IL-1β (lower panel). Scale bar: 50 µm. (M) The percentages of γ-H2A.X positive nuclei were calculated (n=6). (N) The percentages of IL-1β positive area were calculated (n=6). Data were mean ± SEM. *P < 0.05, **P < 0.01, ***P < 0.001 and ****P < 0.0001. One-way ANOVA followed by Tukey's post-test (B, C, E, G, H, J, K, M and N).

The protective effect of Remodelin was further verified in Cisplatin-induced AKI model in which Remodelin was applied before AKI model establishment (Fig. 7A). Scr and BUN measurements indicated that preventive application of Remodelin improved kidney function in mice with AKI (Fig. 7B). Assessment of NGAL mRNA and protein levels revealed that Remodelin application attenuated the injury of renal tubular epithelial cells (Fig. 7C-F and 7H). Furthermore, H&E staining and tubular injury scores demonstrated that Remodelin reduced tubular necrosis, dilation and cast formation (Fig. 7F and 7G). Evaluation of senescence markers showed that preconditioning with Remodelin downregulated the expression of p53 and p21 and reduced the levels of SASP (Fig. 7I-L and 7N). Western blot and IHC results showed a decrease in γ-H2A.X level, indicating that Remodelin mitigated Cisplatin-induced DNA damage in the kidney (Fig. 7I, 7J, 7L and 7M).

### NAT10 interacted with DDX17 to regulate its expression

**Figure 8:**
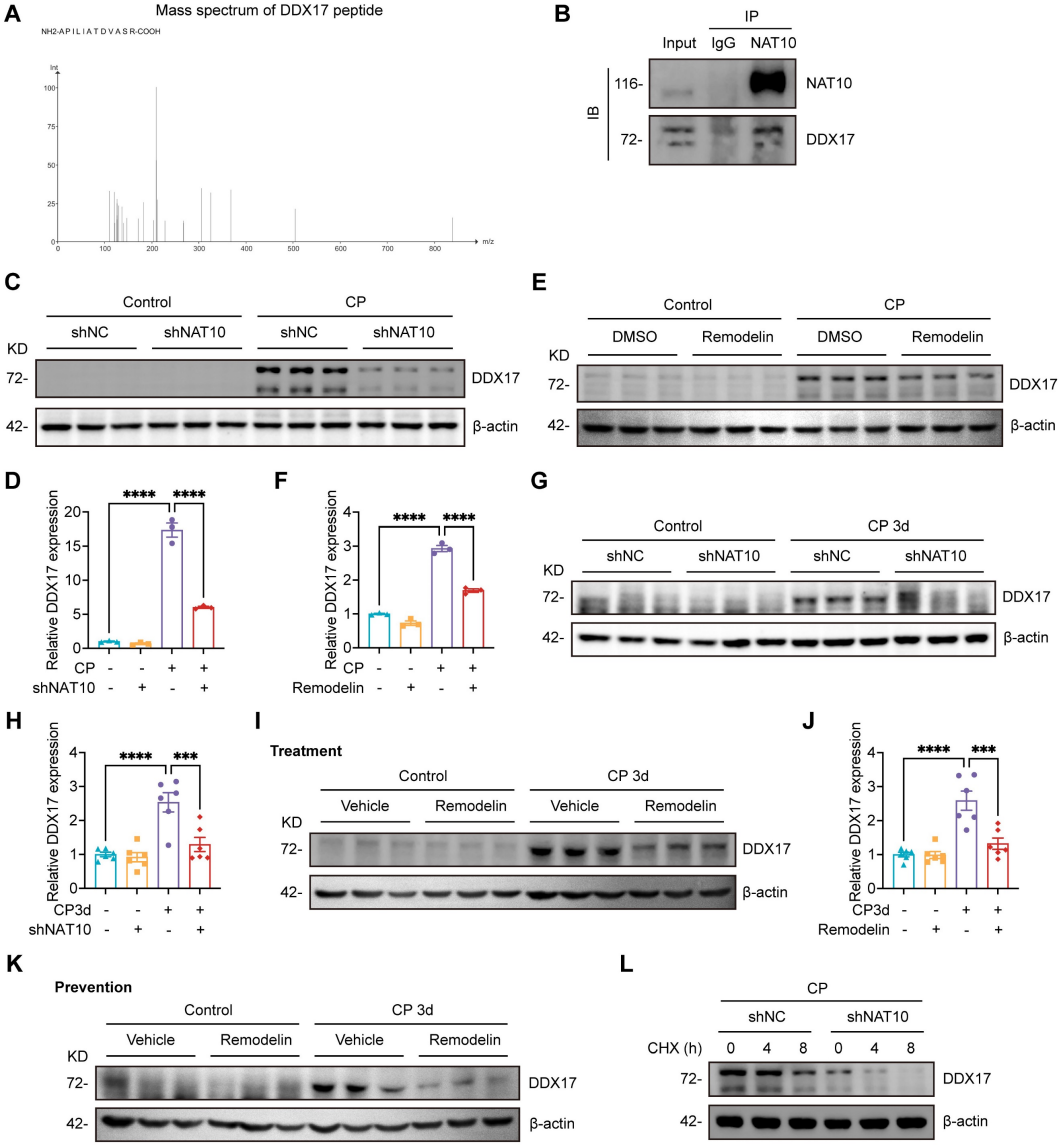
** NAT10 interacted with DDX17 to regulate its expression.** (A) Peptide information of DDX17 identified by mass spectrometry. (B) Co-immunoprecipitation of NAT10 in HK-2 cells. NAT10 and DDX17 were detected by western blot. (C and D) Representative western blot images and quantification of DDX17 in Cisplatin-treated HK-2 cells transfected with negative control lentivirus (shNC) or NAT10 knockdown lentivirus (shNAT10) (n=3). (E and F) Representative western blot images and quantification of DDX17 in Cisplatin-induced HK-2 cells treated with DMSO or Remodelin (n=3). (G and H) Representative western blot images and quantification of DDX17 in the kidney cortex of Cisplatin-induced mice with renal cortex injection of shNC or shNAT10 lentivirus (n=6). (I and J) Representative western blot images and quantification of DDX17 in the kidney cortex of Cisplatin-induced mice treated with vehicle or Remodelin (n=6). (K) Representative western blot images of DDX17 in the kidney cortex of Cisplatin-induced mice with prevention treatment of vehicle or Remodelin. (L) Representative western blot images of DDX17 in Cisplatin-induced HK-2 cells transfected with shNC or shNAT10 lentivirus and then treated with cycloheximide (CHX) for up to 8 hours (n=3). Data were mean ± SEM. ***P < 0.001 and ****P < 0.0001. One-way ANOVA followed by Tukey's post-test (D, F, H and J).

Considering that NAT10 was initially identified as a protein acetyltransferase^35^, ^36^ and the role in Cisplatin-induced AKI was not yet clear, we intended to discover potential NAT10-binding proteins that mediated the effects of NAT10 on Cisplatin-induced AKI. Therefore, we performed immunoprecipitation-coupled mass spectrometry (IP-MS) using kidney cortex from mice. The IP-MS results revealed the binding interaction between NAT10 and DDX17 (Fig. 8A). This interaction was further confirmed *in vitro* through co-immunoprecipitation (Co-IP) assays in HK-2 cells (Fig. 8B). Based on these findings, we investigated whether NAT10 binding influenced DDX17 protein expression. Knockdown of NAT10 downregulated DDX17 protein level in Cisplatin-treated HK-2 cells (Fig. 8C and 8D). Similarly, treatment with Remodelin, also led to a reduction in DDX17 expression (Fig. 8E and 8F). As for *in vivo* experiments, knockdown of NAT10 in the kidney resulted decreased DDX17 protein expression in Cisplatin-induced AKI (Fig. 8G and 8H). Moreover, both therapeutic and preventive application of Remodelin in Cisplatin-treated mice significantly reduced DDX17 protein levels in kidney cortex (Fig. 8I-K and Fig. S3A). We next performed a cycloheximide-chase experiment to determine whether NAT10 affected DDX17 protein stability. NAT10 knockdown accelerated the degradation of DDX17 protein in Cisplatin-induced HK-2 cells (Fig. 8L and Fig. S3B). These results suggested that NAT10 not only interacted with DDX17 but also positively regulated its protein expression under pathological conditions, highlighting a potential regulatory mechanism involved in Cisplatin-induced AKI.

### NAT10-induced cellular injury and senescence was dependent on DDX17

**Figure 9:**
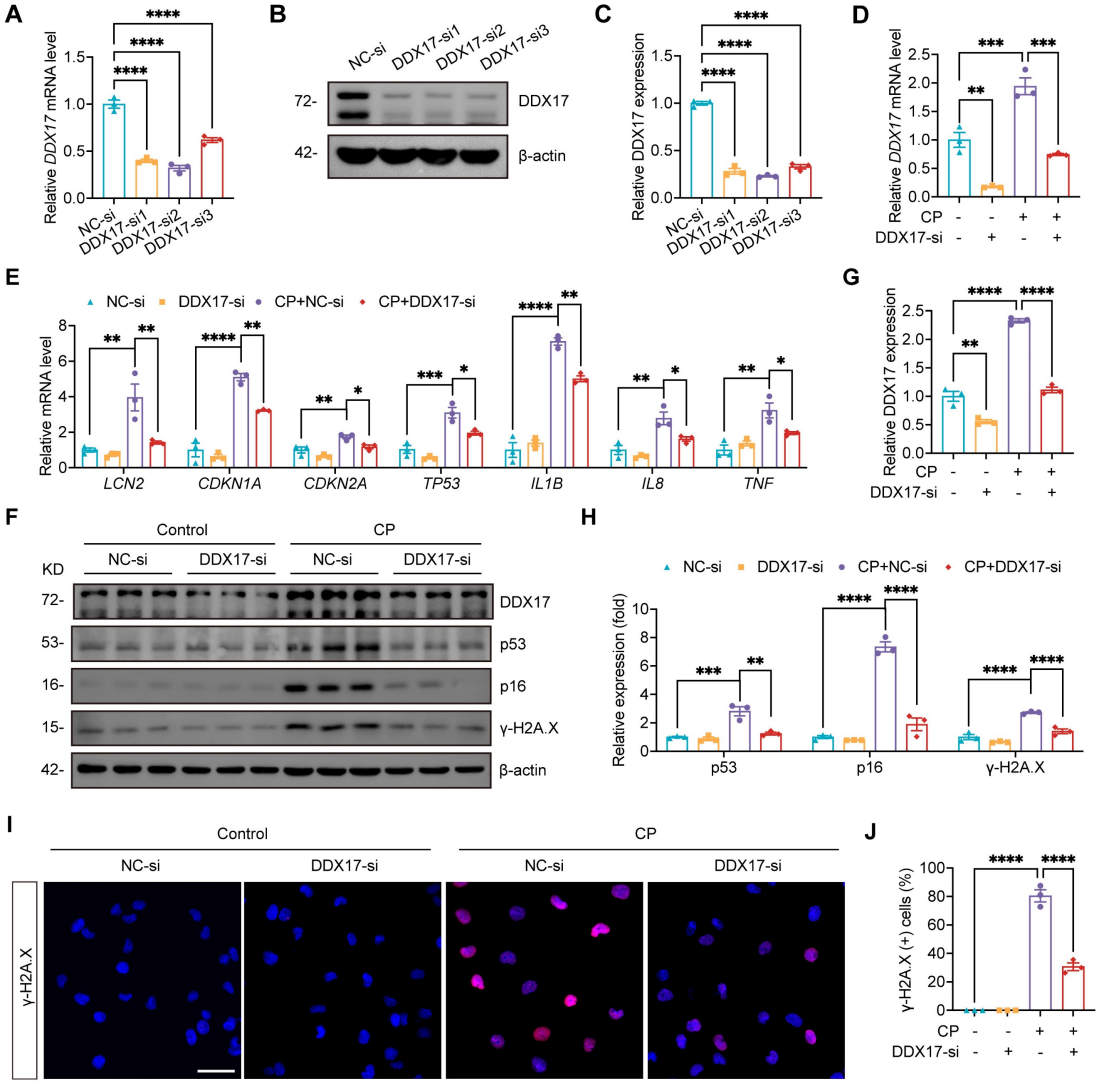
** Silencing DDX17 attenuated Cisplatin-induced injury and cellular senescence in HK-2 cells.** (A) qPCR analysis of *DDX17* in HK-2 cells transfected with negative control siRNA (NC-si) or DDX17 siRNA (DDX17-si) (n=3). (B and C) Representative western blot images and quantification of DDX17 in HK-2 cells (n=3). (D and E) qPCR analysis of *DDX17*, *LCN2*, *CDKN1A*, *CDKN2A*, *TP53*, *IL1B*, *IL8* and *TNF* in Cisplatin-induced HK-2 cells transfected with NC-si or DDX17-si (n=3). (F-H) Representative western blot images and quantification of DDX17, p53, p16 and γ-H2A.X in HK-2 cells (n=3). (I) Representative immunofluorescence staining images of γ-H2A.X in HK-2 cells. Scale bar: 50 µm. (J) The percentages of γ-H2A.X positive cells were calculated (n=3). Data were mean ± SEM. *P < 0.05, **P < 0.001, ***P < 0.001 and ****P < 0.0001. One-way ANOVA followed by Tukey's post-test (A, C-E, G, H and J).

Our study has established that NAT10 regulated DDX17 expression in AKI. However, the role of DDX17 in Cisplatin-induced AKI and cellular senescence remained unclear. To investigate this, we silenced DDX17 in HK-2 cells using siRNA and selected the most efficient siRNA (DDX17-si2) for subsequent experiments (Fig. 9A-C). The qPCR analysis revealed that DDX17 silencing significantly suppressed the upregulation of *LCN2* mRNA induced by Cisplatin, indicating amelioration of injury in HK-2 cells (Fig. 9D and 9E). Furthermore, silencing DDX17 downregulated the expression of senescence markers and reduced mRNA levels of SASP factors (Fig. 9E-H). Western blot and IF results also demonstrated a notable reduction in γ-H2A.X levels, confirming attenuation of DNA damage in Cisplatin-treated HK-2 cells upon DDX17 silencing (Fig. 9F and 9H-J). Together, these results indicated that DDX17 silencing alleviated Cisplatin-induced cellular damage and senescence in HK-2 cells, underscoring the critical role of DDX17 in the pathogenesis of AKI.

**Figure 10:**
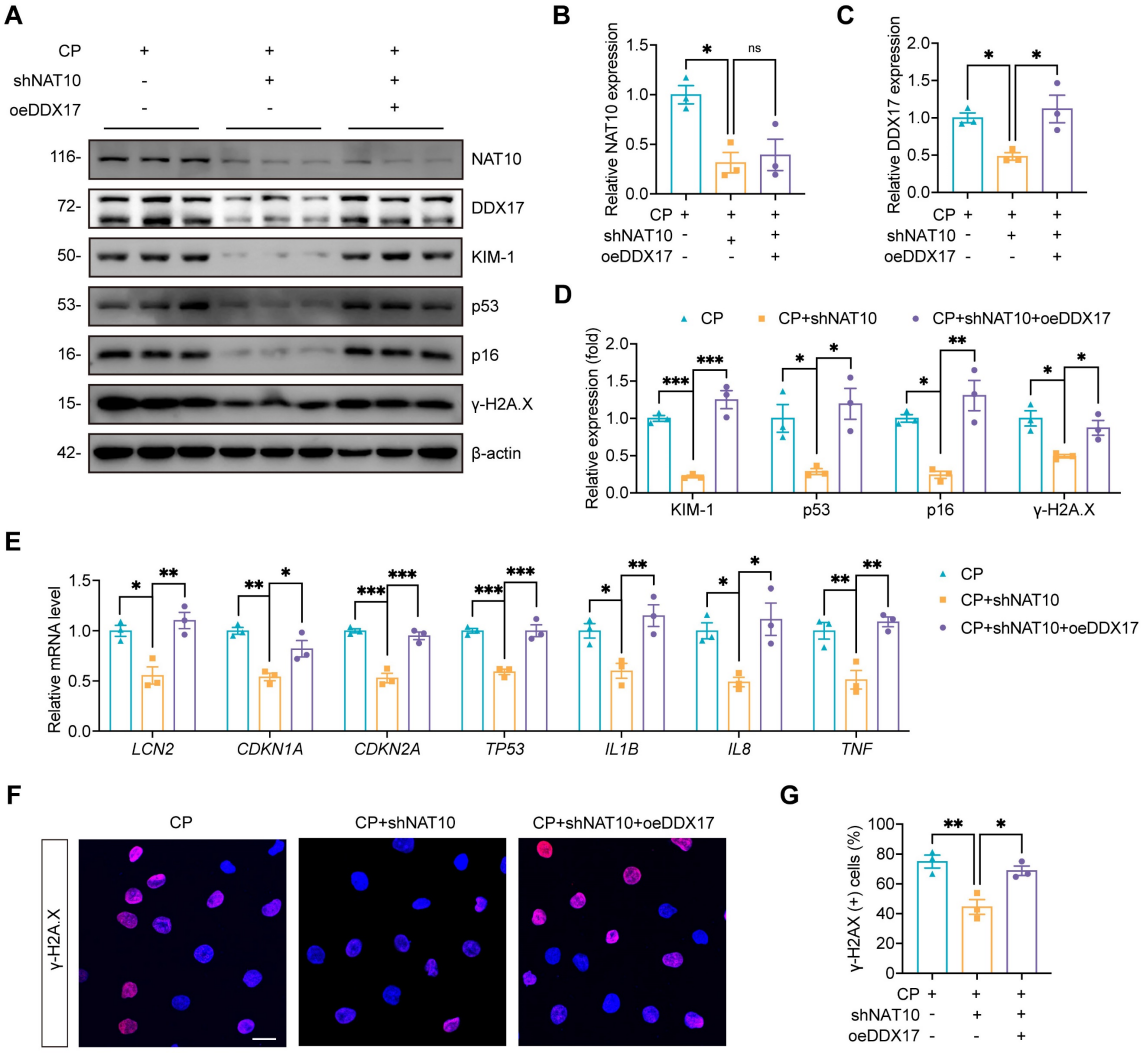
** NAT10-induced cellular injury and senescence was dependent on DDX17.** (A-D) Representative western blot images and quantification of NAT10, DDX17, KIM-1, p53, p16 and γ-H2A.X in Cisplatin-induced HK-2 cells transfected with NAT10 knockdown lentivirus (shNAT10) and DDX17 overexpression plasmids (oeDDX17) (n=3). (E) qPCR analysis of *LCN2*, *CDKN1A*, *CDKN2A*, *TP53*, *IL1B*, *IL8* and *TNF* in HK-2 cells (n=3). (F) Representative immunofluorescence staining images of γ-H2A.X in HK-2 cells. Scale bar: 25 µm. (G) The percentages of γ-H2A.X positive cells were calculated (n=3). Data were mean ± SEM. *P < 0.05, **P < 0.01 and ***P < 0.001. ns, not significant. One-way ANOVA followed by Tukey's post-test (B-E and G).

We further investigated whether the effects of NAT10 on Cisplatin-induced tubular injury and senescence was dependent on DDX17. In Cisplatin-treated HK-2 cells, we performed NAT10 knockdown using lentivirus and DDX17 overexpression via plasmid transfection. Western blot analysis confirmed efficient overexpression of DDX17, which did not affect the protein level of NAT10 (Fig. 10A-C). Assessment of tubular injury and cellular senescence markers revealed that DDX17 overexpression abrogated the protective effects conferred by NAT10 knockdown (Fig. 10A and 10D-G). A recent study reported that NAT10 promoted podocyte senescence by regulating TLR2 in adriamycin-induced nephropathy^23^. Therefore, we considered whether TLR2 was also involved in NAT10-mediated regulation of AKI. The qPCR and western blot analyses showed that the expression of TLR2 was significantly increased in Cisplatin-induced mice and HK-2 cells, but NAT10 knockdown did not significantly alter the expression of TLR2 (Fig. S4). In summary, our findings showed that NAT10 induced tubular injury and senescence was dependent on DDX17.

## Discussion

**Figure 11:**
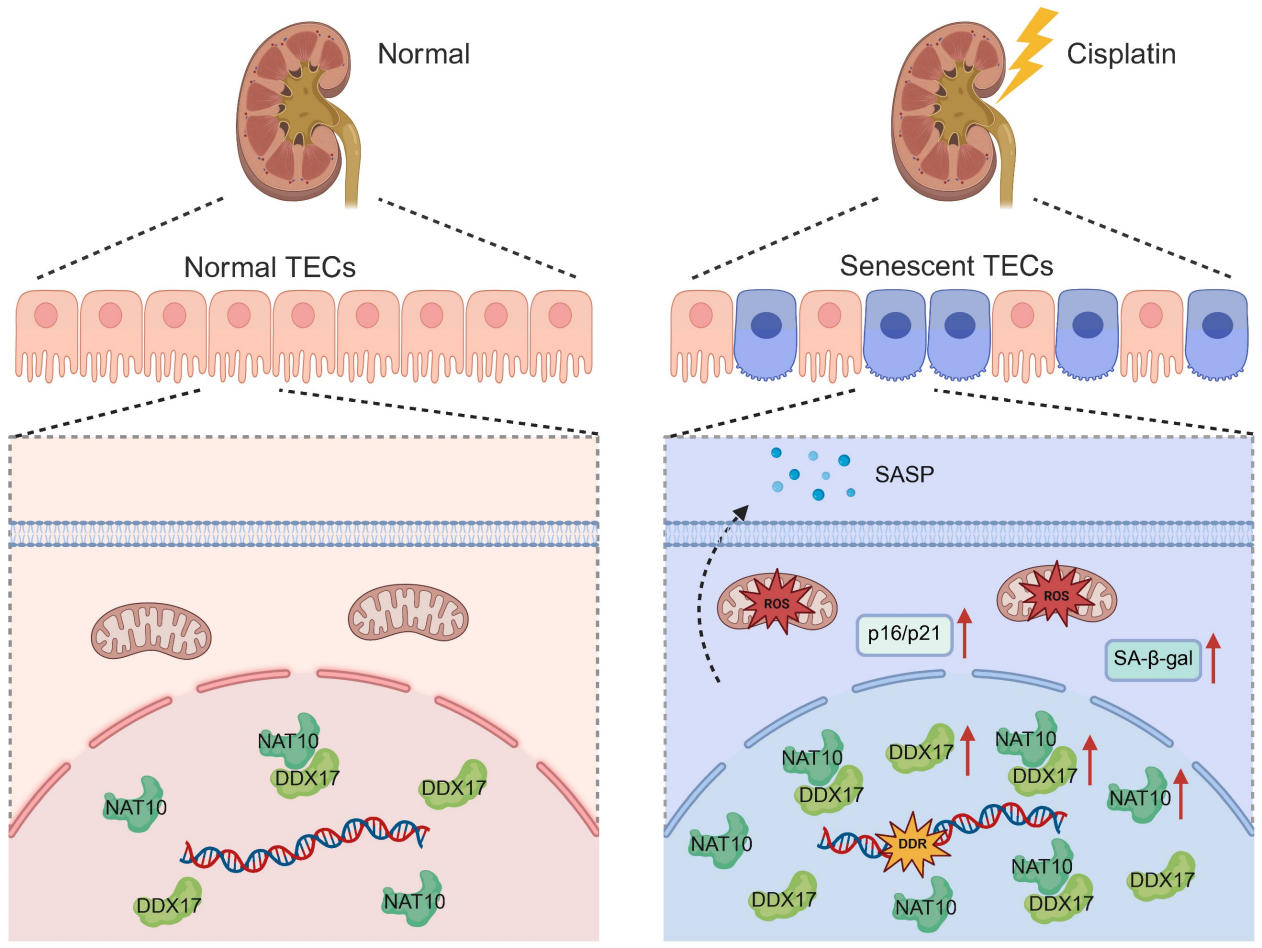
Schematic diagram illustrating that NAT10 interacted with DDX17 to regulate Cisplatin-induced tubular epithelial cell senescence. DDR, DNA damage response. SA-β-gal, senescence-associated-β-galactosidase.

In the current study, we unveiled that NAT10 promoted Cisplatin-induced AKI through the induction of cellular senescence in tubular epithelial cells. Lentivirus mediated knockdown or pharmacological inhibition of NAT10 significantly attenuated renal dysfunction, cellular damage, and senescence phenotypes. Importantly, we identified DDX17 as a novel downstream effector of NAT10. And NAT10 contributed to AKI via a pathway involving DDX17-mediated cellular senescence (Figure 11).

Cellular senescence of renal tubular epithelial cells has emerged as a pivotal mechanism contributing to the pathogenesis and progression of AKI, leading to maladaptive repair and CKD^13^. Upon Cisplatin-induced AKI, renal tubular epithelial cells undergo stress-induced senescence characterized by cell cycle arrest via p53/p21 and p16/Rb pathways, elevated activity of SA-β-gal, and secretion of pro-inflammatory and pro-fibrotic factors known as the SASP^11^, ^12^. Evidence from our study, utilizing both Cisplatin-induced animal model and cell model, also confirmed cellular senescence of tubular epithelial cells in AKI. However, the role and underlying regulatory mechanisms of tubular epithelial cell senescence in AKI remain poorly understood.

NAT10 plays critical roles in multiple cellular processes, such as cell mitosis, DNA damage response, autophagy and apoptosis^15^. Accumulating evidence has demonstrated that NAT10 is closely associated with the pathogenesis of cancer, cardiovascular disorders, liver fibrosis and *etc*^18^, ^37^, ^38^. In AKI, NAT10 has been shown to aggravate renal inflammation by enhancing the stability of *CCL2* and *CXCL1* mRNAs through ac4C modification^20^. Our study provided evidence for the first time, that knockdown of NAT10 in the kidney ameliorated Cisplatin-induced renal dysfunction and cellular senescence. Both therapeutic and preventive application of the NAT10 inhibitor Remodelin significantly attenuated renal injury and senescence in mice with AKI. Furthermore, prolonged treatment with Remodelin for up to one month did not induce functional impairment or abnormal gross morphology in the kidney, liver, heart or spleen, supporting its favorable safety and therapeutic potential. By applying the senomorphic drug Ruxolitinib, our study also confirmed that NAT10 promoted Cisplatin-induced AKI via accelerating the senescence of tubular epithelial cells.

Whereas considerable efforts have been devoted to elucidating NAT10's role in ac4C-dependent mRNA modification, understanding of its protein-protein interactions remains notably limited. Our study provided evidence of the interaction between NAT10 and DDX17 through IP-MS combined with Co-IP assay for the first time. Both *in vivo* and *in vitro* experiments consistently demonstrated that either knockdown of NAT10 or treatment with Remodelin downregulated DDX17 protein expression. These findings firstly confirmed that NAT10 regulated DDX17 protein expression through protein-protein interaction in Cisplatin-induced AKI. More importantly, our results indicated that NAT10 enhanced the protein stability of DDX17 to regulate its expression. Previous studies have reported that NAT10 functioned as a protein acetyltransferase^35^, ^36^. However, whether NAT10 influences DDX17 protein stability through direct acetylation remains to be explored.

A recent bioinformatics analysis based on GEO database proposed DDX17 as a potential biomarker for AKI^39^. Our study revealed that DDX17 was upregulated in AKI, and silencing DDX17 suppressed Cisplatin-induced damage and cellular senescence in HK-2 cells. These findings not only confirmed DDX17 as a contributor to AKI but also uncovered, for the first time, its novel role in regulating stress-induced cellular senescence. Besides, we also verified that NAT10-induced cellular injury and senescence was dependent on DDX17 *in vitro*. A recent study reported that NAT10 mediated TLR2 to promote podocyte senescence in adriamycin-induced nephropathy^23^. But we didn't detect any changes in TLR2 expression after NAT10 knockdown or inhibition, suggesting that the NAT10/TLR2/senescence signaling pathway may primarily play a role in adriamycin-induced nephropathy.

Several limitations of this study should be acknowledged. Although mouse models and human tubular cell lines provided robust evidence supporting the role of NAT10 in AKI and cellular senescence, validation in human kidney tissues would enhance the clinical relevance of these findings. Despite the employment of NAT10 knockdown lentivirus and Remodelin *in vivo* to intervene in the expression and function of NAT10 in tubular epithelial cells, we acknowledged that immune cells and other parenchymal cells in the kidney may also be affected. Future studies applying tubule-specific *Nat10* conditional knockout mice will more precisely define the role of NAT10 in Cisplatin-induced tubular epithelial cell injury and senescence. In addition, the effects of DDX17 in renal dysfunction and cellular senescence remains to be validated in AKI animal models in future studies.

In conclusion, this study systematically demonstrated that NAT10 promoted Cisplatin-induced AKI and renal tubular senescence. Mechanistically, we identified DDX17 as a NAT10 interacting protein and revealed a novel NAT10/DDX17 pathway contributing to cellular senescence in AKI. Collectively, our study highlighted NAT10 as a potential therapeutic target for AKI.

## Supplementary Material

Supplementary figures and table 1.

Supplementary table 2.
