# Supplementary material for: NAT10 Promotes Tubular Epithelial Cell Senescence in Cisplatin-Induced Acute Kidney Injury by Regulating DDX17
Source: Int J Biol Sci. 2026 Jan 30;22(4):2177–95. doi: 10.7150/ijbs.127909 (PMC12905645; doi:10.7150/ijbs.127909)
Supplement: Supplementary file 1 — Supplementary figures and table 1. [file ijbsv22p2177s1.pdf]

Supplementary Materials

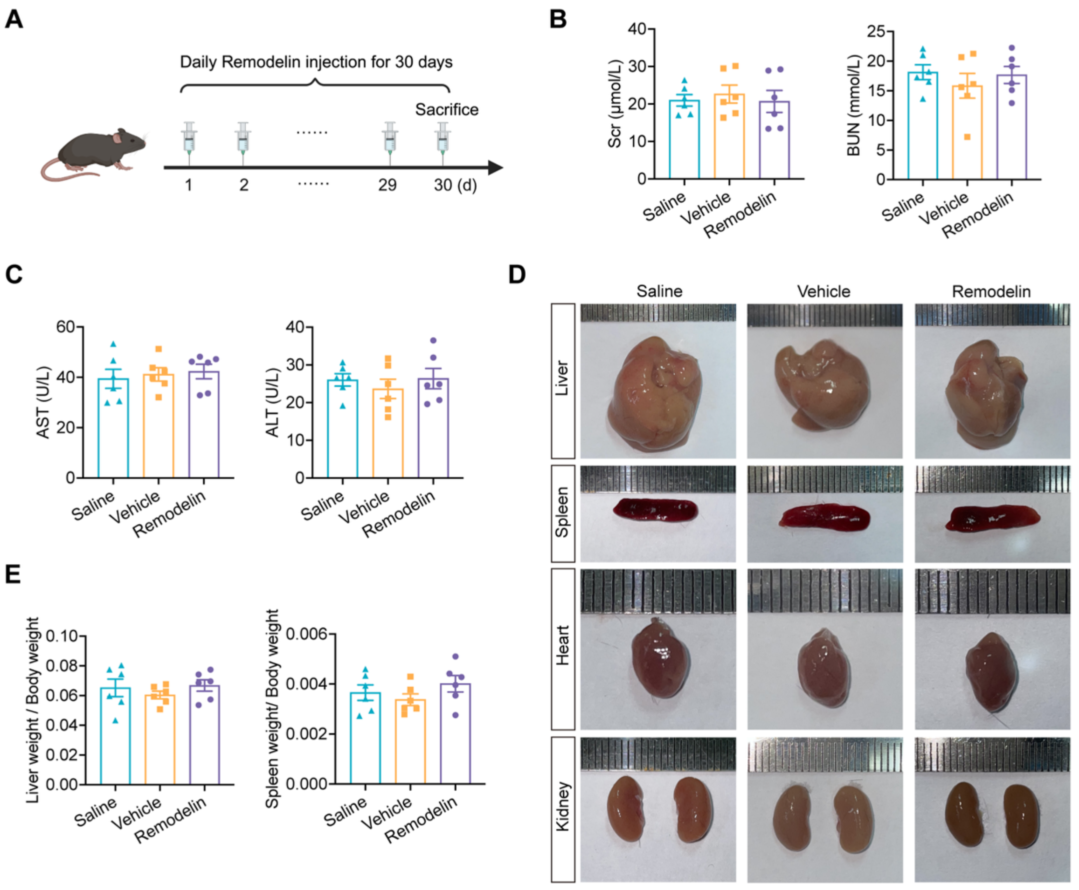

Figure S1. Safety and toxicity of Remodelin in mice

(A) Schematic diagram of Remodelin administration via intraperitoneal injection for 1 month in mice. (B) Serum creatinine (Scr) and blood urea nitrogen (BUN) in mice (n=6). (C) Serum aspartate aminotransferase (AST) and alanine aminotransferase (ALT) levels in mice (n=6). (D) Liver, spleen, heart and kidney morphology of mice. (E) The ratios of liver weight to body weight and spleen weight to body weight (n=6).

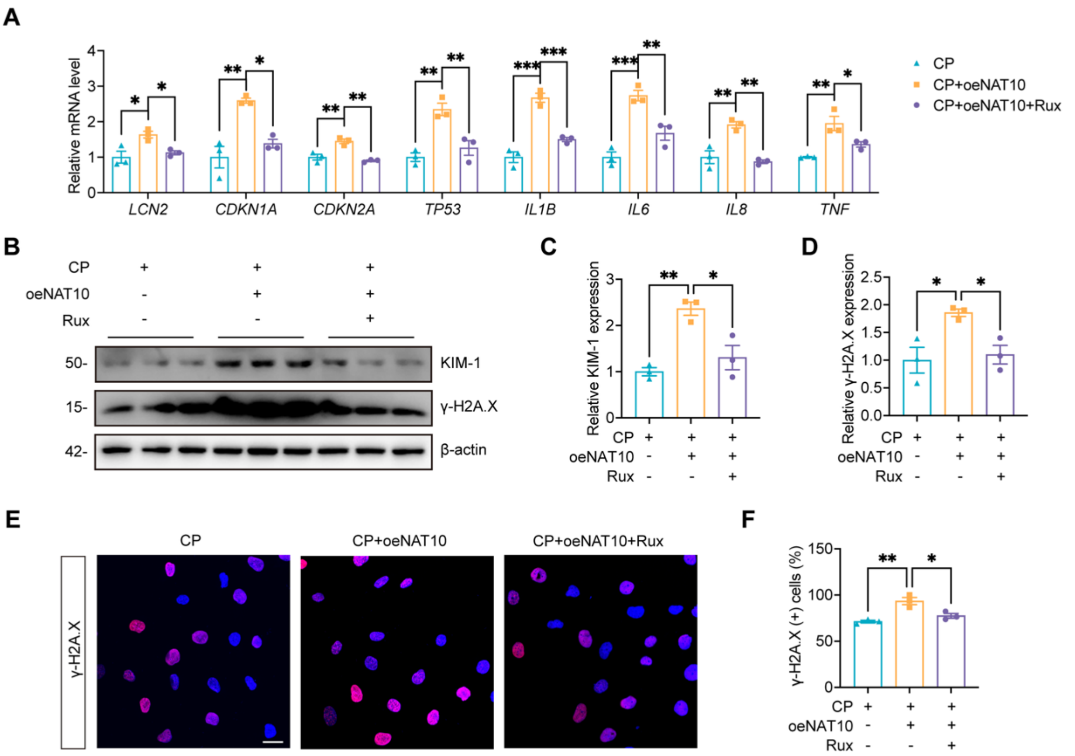

Figure S2. Ruxolitinib treatment ameliorated cellular injury and senescence caused by NAT10 overexpression in Cisplatin-induced HK-2 cells

(A) qPCR analysis of *LCN2*, *CDKN1A*, *CDKN2A*, *TP53*, *IL1B*, *IL6*, *IL8* and *TNF* in Cisplatin-induced HK-2 cells transfected with NAT10 overexpressing plasmids (oeNAT10) or treated with Ruxolitinib (Rux) (n=3). (B-D) Representative western blot images and quantification of KIM-1 and γ-H2A.X in HK-2 cells (n=3). (E) Representative immunofluorescence staining images of γ-H2A.X in HK-2 cells. Scale bar: 25 μm. (F) The percentages of γ-H2A.X positive cells were calculated (n=3). Data were mean ± SEM. **P* < 0.05, ***P* < 0.01 and ****P* < 0.001. One-way ANOVA followed by Tukey's post-test (A, C, D and F).

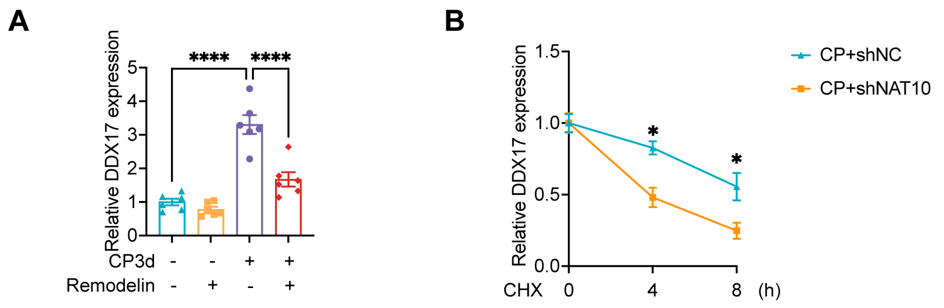

Figure S3. Remodelin treatment downregulated the expression of DDX17 and NAT10 knockdown reduced the protein stability of DDX17

(A) Quantification of DDX17 immunoblots in the kidney cortex of Cisplatin-induced mice with prevention treatment of vehicle or Remodelin (n=6). (B) Quantification of DDX17 immunoblots in Cisplatin-induced HK-2 cells transfected with negative control lentivirus (shNC) or NAT10 knockdown lentivirus (shNAT10) and then treated with cycloheximide (CHX) for up to 8 hours (n=3). Data were mean ± SEM. *$P < 0.05$ and ****$P < 0.0001$. One-way ANOVA followed by Tukey's post-test (A). Two-tailed unpaired Student t test (B).

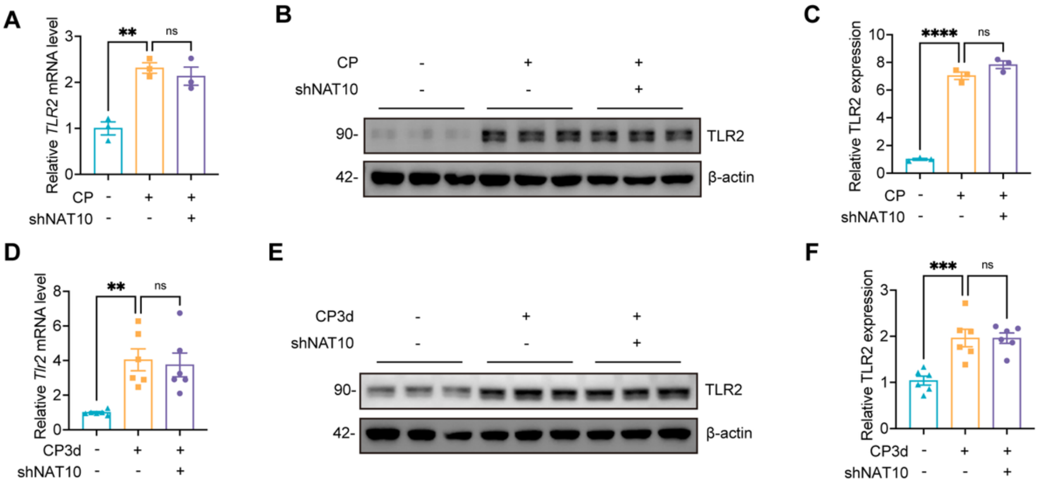

Figure S4. NAT10 knockdown did not significantly alter the expression of TLR2

(A) qPCR analysis of *TLR2* in Cisplatin-induced HK-2 cells transfected with NAT10 knockdown lentivirus (shNAT10) (n=3). (B and C) Representative western blot images and quantification of TLR2 in HK-2 cells (n=3). (D) qPCR analysis of *Tlr2* in the kidney cortex of Cisplatin-induced mice with kidney cortex injection of shNAT10 lentivirus (n=6). (E and F) Representative western blot images and quantification of TLR2 in the kidney cortex (n=6). Data were mean ± SEM. $^{**}P < 0.01$, $^{***}P < 0.001$ and $^{****}P < 0.0001$. ns, not significant. One-way ANOVA followed by Tukey's post-test (A, C, D and F).

Table S1. Primer sequences used in Real-time quantitative PCR

Gene	Forward primer (5'-3')	Reverse primer (3'-5')
Nat10	TCAACATCACCCGCATCGTT	GGTCGTTGGGAGAGTTCTTG
Actb	CCACCATGTACCCAGGCATT	CGGACTCATCGTACTCCTGC
Lcn2	ACCACGGACTACAACCAGTTC	CAGCTCCTTGGTTCTT CCATAC
Cdkn1a	TCCAGACATTCAGAGCCACAG	AAAGTTCC ACCGTTCTCGGG
Tp53	TCCGAAGACTGGATGACTGC	GATC GTCCATGCAGTGAGGT
Il6	TGCCTTCTTGGGACTGATG	ACTCTGGCTTTGTCTTTCTTGT
Il1b	TGCCACCTTTTGACAGTGATG	ATGTGCTGCTGCGAGATTTG
Tnf	GTAGCCCACGTCGTAGCAAA	ACAAGGTACAACCCATCGGC
Tlr2	AACCTCAGACAAAGCGTCAAAT	ATCACACACCCCAGAAGCAT
NAT10	ATTCACACCGTAAGCAGCGA	CAGAACTTGAGGAGCCTGGG
ACTB	CTCACCATGGATGATGATATCGC	AGGAATCCTTCTGACCCATGC
LCN2	CTGAGTGCACAGGTGCCG	TTTAGCAGACAAGGTGGGGC
CDKN1A	TGGACCTGTCACTGTCTTGT	AGGCAGAAGATGTAGAGCGG
CDKN2A	ACTTCAGGGGTGCCACATTC	CGACCCTGTCCCTCAAATCC
TP53	GCCCCTCCTCAGCATCTTAT	AAAGCT GTTCCGTCCCAGTA
IL1B	TTCGAGGCACAAGGCACAA	AGTCATCCTCATTGCCACTGTAA
IL6	CCCACCGGGAACGAAAGA	TGGACCGAAGGCGCTTGT
IL8	GGAGAAGTTTTTGAAGAGGGCTG	ACAGACCCACACAATACATGAAG
TNF	TCTCCTTCCTGATCGTGGCA	CAGCTTGAGGGTTTGCTACAAC
DDX17	AGTGCTGGAAGAGGCCAATC	GAGAACGACCACCCCCG
TLR2	GTTCTCTCAGGTGACTGCTC	CCCTGTCTTCCTGCCTTCAC
